# Structural changes in femoral bone microstructure of female rabbits after intramuscular administration of quercetin

**DOI:** 10.1186/s13028-016-0225-4

**Published:** 2016-06-29

**Authors:** Ramona Babosova, Hana Duranova, Radoslav Omelka, Veronika Kovacova, Maria Adamkovicova, Birgit Grosskopf, Marcela Capcarova, Monika Martiniakova

**Affiliations:** 1 Department of Zoology and Anthropology, Constantine the Philosopher University, 949 74 Nitra, Slovakia; 2 Department of Botany and Genetics, Constantine the Philosopher University, 949 74 Nitra, Slovakia; 3 Institute of Zoology and Anthropology, Georg-August University, 37 073 Göttingen, Germany; 4 Department of Animal Physiology, Slovak University of Agriculture, 949 76 Nitra, Slovakia

**Keywords:** Bone microstructure, Histomorphometry, Quercetin, Rabbit, Intramuscular administration

## Abstract

**Background:**

Quercetin is one of the best known flavonoids being present in a variety of fruits and vegetables. It has cardioprotective, anticarcinogenic, antioxidant, anti-inflammatory and antiapoptotic properties. Some studies suggest that quercetin has protective effects on bone. However, its influence on qualitative and quantitative histological characteristics of compact bone is still unknown. In our study, 12 clinically healthy five-month-old female rabbits were divided into four groups of three animals each. Quercetin was applied intramuscularly in various concentrations; 10 µg/kg body weight (bw) in the E1 group, 100 µg/kg bw in the E2 group, and 1000 µg/kg bw in the E3 group for 90 days, 3 times per week. Three rabbits without exposure to quercetin served as a control (C) group. Differences in femoral bone microstructure among groups were evaluated.

**Results:**

Qualitative histological characteristics of compact bone differed between rabbits from the E1 and E2 groups. Primary vascular longitudinal bone tissue was not found in some areas near the endosteal surface due to increased endocortical bone resorption. In addition, periosteal border of rabbits from the E1 group was composed of a thicker layer of primary vascular longitudinal bone tissue than in the other groups. In all groups of rabbits administered quercetin, a lower density of secondary osteons was observed. Histomorphometrical evaluations showed significantly decreased sizes of the primary osteons’ vascular canals in individuals from the E1 and E2 groups. Secondary osteons were significantly smaller in rabbits from the E1, E2, E3 groups when compared to the C group. Cortical bone thickness was significantly increased in females from the E1 and E2 groups.

**Conclusions:**

The results indicate that quercetin has not only a positive dose–response on qualitative and quantitative histological characteristics of the compact bone of female rabbits as it would be expected.

## Background

Flavonoids belong to the group of polyphenolic secondary herbal substances that have beneficial effects on human health [1]. Quercetin (2-(3,4-dihydroxyphenyl)-3,5,7-trihydroxy-4H-1-benzopyran-4-one; 3,3’,4’,5,7-pentahydroxyflavone) is one of the best characterized flavonoids present in fruits and vegetables [2]. It has cardioprotective [3], anticarcinogenic [4], antioxidant [5], anti-inflammatory [6] and antiapoptotic properties [7]. It also protects against reactive oxygen species (ROS) and reactive nitrogen species (RNS) [8, 9]. Some studies [10, 11] suggest that quercetin also has protective effects on bone as it inhibits bone loss by affecting osteoclastogenesis. Horcajada–Molteni et al. [12] and Tsuji et al. [13] state that quercetin and its dietary analogue rutin inhibit osteopenia in ovariectomized rats. On the other hand, quercetin stimulates differentiation of osteoblasts and MG-63 osteoblast-like cells in rats [14]. Besides its beneficial health effects, potentially toxic actions of quercetin related to mutagenicity, prooxidant activity, mitochondrial toxicity, and inhibition of key enzymes involve in hormone metabolism have been demonstrated [15, 16]. However, the impact of quercetin on qualitative and quantitative histological characteristics of the compact bone is still unknown. Therefore, the aim of this study was to investigate femoral bone microstructure of adult female rabbits after intramuscular application of quercetin.

## Methods

### Animals

The study was conducted on 12 clinically healthy adult female rabbits of meat line M91, maternal albinotic line (crossbreed New Zealand White, Buskat rabbit, French Silver) and paternal acromalictic line (crossbreed Nitra’s rabbit, Californian rabbit, Big Light Silver) of approximately 5 months of age, with a body weight (bw) of 4.00 ± 0.5 kg. Animals were obtained from an experimental farm of the Animal Production Research Centre in Nitra (Slovak Republic) and were housed in individual flat-deck wire cages under a constant photoperiod of 12 h of daylight, temperature 20–24 °C and humidity 55 ± 10 %, with an access to food (feed mixture) and drinking water ad libitum. Adult female rabbits were randomly divided into four groups of three animals each: E1, E2, E3 and C. The rabbits from the E1, E2 and E3 groups were intramuscularly injected with quercetin (Sigma-Aldrich, Germany) at doses of 10, 100 and 1000 μg/kg bw, respectively for 90 days, 3 times per week. The doses of quercetin (reflecting the natural exposure of animals to quercetin in rabbit feed) were chosen based on literature data [17–19]. Rabbits from the C group (controls) were injected by a saline solution at the same time for 90 days. In general, the rabbits were kept for other investigations (e.g. histological and biochemical analyses) at the Animal Production Research Centre in Nitra. The present study was performed as an additional investigation focused on compact bone microstructure.

### Procedures

At the end of experimental period (i.e. after 90 days), all the rabbits were killed and their femurs were used for analyses. For histological evaluation, the right femurs were sectioned at the diaphyseal midshaft and the segments were fixed in HistoChoice fixative (Amresco, USA). The segments were then dehydrated in increasing grades (40–100 %) of ethanol and embedded in Biodur epoxy resin (Günter von Hagens, Heidelberg, Germany) as previously described [20]. Transverse sections (70–80 μm) were prepared with a sawing microtome (Leitz 1600, Leica, Wetzlar, Germany) and fixed onto glass slides by Eukitt (Merck, Darmstadt, Germany) [21]. The qualitative histological characteristics of the compact bone were determined according to the internationally accepted classification systems of Enlow and Brown [22] and de Ricqlés et al. [23], who classify bone tissue into three main categories: primary vascular tissue, non-vascular tissue and Haversian bone tissue. Various patterns of vascularization occur in primary vascular bone tissue: longitudinal, radial, reticular, plexiform, laminar, lepidosteoid, acellular, fibriform and protohaversian. Three subcategories in Haversian bone tissue are known: irregular, endosteal and dense. The quantitative (histomorphometrical) variables were assessed using the software Motic Images Plus 2.0 ML (Motic China Group Co., Ltd.). We measured area, perimeter, minimum and maximum diameters of primary osteons’ vascular canals, Haversian canals, and secondary osteons in the four cross-sectional quadrants (i.e. anterior, posterior, medial and lateral) to minimize inter-animal differences. The diaphyseal cortical bone thickness was also measured by Motic Images Plus 2.0 ML software. Twenty random areas were selected and average thickness was calculated for each femur.

### Statistics

Statistical analysis was performed using SPSS 8.0 software. All data were expressed as mean ± standard deviation (SD). The unpaired Games-Howell’s test was used for establishing statistical significance between all groups.

## Results

### Qualitative histological analysis

**Fig. 1:**
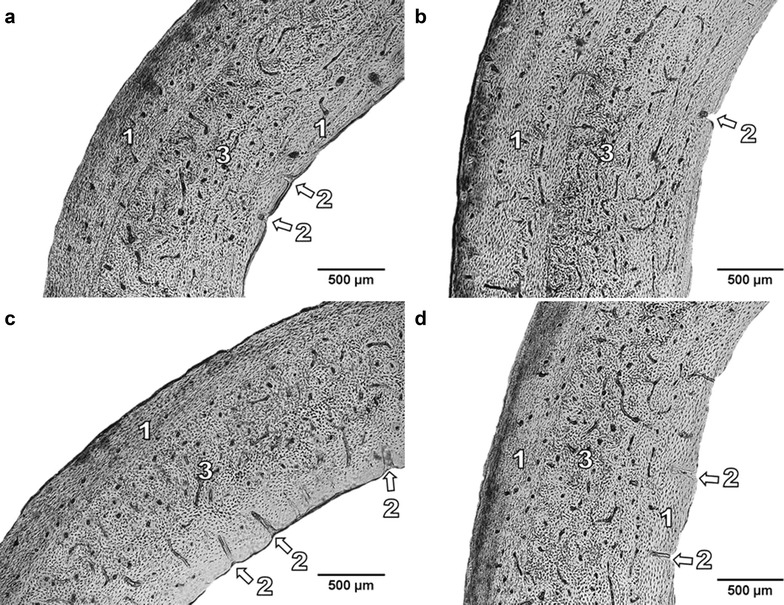
**a**, **d** Photomicrographs showing the structure of the compact bone of rabbits from the C and E3 groups. *1* Primary vascular longitudinal bone tissue. *2* Primary vascular radial bone tissue. *3* Dense Haversian bone tissue. **b**, **c** Photomicrographs showing the structure of the compact bone in rabbits from the E1 and E2 groups. *1* Primary vascular longitudinal bone tissue. *2* Primary vascular radial bone tissue. *3* Irregular Haversian bone tissue

The periosteal and endosteal surfaces of femurs in rabbits from the C group were formed by primary vascular longitudinal bone tissue. This tissue included vascular canals, which ran in a direction essentially parallel to the long axis of the bone. Near endosteal surfaces, primary vascular radial bone tissue (formed by branching or non-branching vascular canals radiating from the marrow cavity) and/or Haversian bone tissues were also found. Haversian bone tissue was characterized by the presence of isolated and scattered secondary osteons (irregular Haversian bone tissue) or by a large density of the osteons (dense Haversian bone tissue). The middle part of the *substantia compacta* was formed by a layer of irregular and/or dense Haversian bone tissues (Fig. 1a).

Rabbits exposed to quercetin displayed differences in compact bone microstructure compared to the C group. In rabbits from the E1 (Fig. 1b) and E2 (Fig. 1c) groups, primary vascular longitudinal bone tissue was not observed in some areas (in anterior and posterior views) near endosteal surfaces. These areas were created by primary vascular radial and/or Haversian bone tissues. The middle part of *substantia compacta* was formed not only by Haversian bone tissue but also by primary vascular longitudinal bone tissue. In rabbits from the E1 group, the periosteal border consisted of a thicker layer of primary vascular longitudinal bone tissue when compared to the other groups. The animals from the E3 (Fig. 1d) group had a compact bone microstructure similar to rabbits from the C group although the number of secondary osteons was lower as found in rabbits from the E1 and E2 groups.

### Quantitative histological analysis

In total, 480 vascular canals of primary osteons, 480 Haversian canals and 480 secondary osteons were measured. The results are summarized in Table 1. All variables (area, perimeter, maximum and minimum diameters) of the primary osteons’ vascular canals were significantly decreased in groups E2 and E3 when compared to the C group. Significant differences were also found between E1 and E2, and E1 and E3 groups (except for minimum diameter). Haversian canals’ values did not differ significantly between groups. On the other hand, secondary osteon values were significantly lower in rabbits from the E1, E2 and E3 groups compared to the C group. Significant differences were also demonstrated between the E1 and E2 groups.

**Table 1:** Data of the primary osteons’ vascular canals, Haversian canals and secondary osteons in adult rabbits from the E1, E2, E3 and C groups

	Rabbits group		N	Area (μm^2^)	Perimeter (μm)	Max. diameter (μm)	Min. diameter (μm)
Vascular canals of primary osteons	E1	(1)	120	399.27 ± 63.02	71.83 ± 5.84	12.57 ± 1.39	10.15 ± 1.02
	E2	(2)	120	358.42 ± 52.11	67.82 ± 4.88	11.77 ± 1.02	9.73 ± 1.00
	E3	(3)	120	359.38 ± 62.33	67.90 ± 5.66	11.75 ± 1.18	9.77 ± 1.14
	C	(4)	120	408.30 ± 79.95	72.22 ± 7.21	12.43 ± 1.45	10.45 ± 1.34
	Games–Howell’s test			1:2^**^; 1:3^**^; 2:4^**^; 3:4^**^	1:2^**^; 1:3^**^; 2:4^**^; 3:4^**^	1:2^**^; 1:3^**^; 2:4^**^; 3:4^**^	1:2^*^; 1:3^**^; 2:4^**^
Haversian canals	E1	(1)	120	329.56 ± 57.64	65.15 ± 5.51	11.33 ± 1.16	9.30 ± 1.17
	E2	(2)	120	317.14 ± 54.53	63.84 ± 5.67	11.08 ± 1.27	9.15 ± 1.04
	E3	(3)	120	318.62 ± 56.46	64.02 ± 5.63	11.18 ± 1.16	9.10 ± 1.07
	C	(4)	120	334.86 ± 69.49	65.47 ± 6.67	11.37 ± 1.35	9.38 ± 1.18
	Games–Howell’s test			*NS*	*NS*	*NS*	*NS*
Secondary osteons	E1	(1)	120	5976.51 ± 2236.50	276.00 ± 51.00	48.79 ± 9.85	38.04 ± 8.15
	E2	(2)	120	4945.24 ± 1691.32	252.24 ± 44.01	44.93 ± 8.60	34.31 ± 7.17
	E3	(3)	120	5355.22 ± 2046.25	262.10 ± 48.34	46.37 ± 9.67	35.97 ± 7.77
	C	(4)	120	6982.93 ± 2773.93	299.10 ± 55.97	53.13 ± 10.65	40.80 ± 9.44
	Games–Howell’s test			1:2^**^; 1:4^*^; 2:4^**^; 3:4^**^	1:2^**^; 1:4^*^; 2:4^**^; 3:4^**^	1:2^**^; 1:4^*^; 2:4^**^; 3:4^**^	1:2^**^; 2:4^**^; 3:4^**^

*N* number of measured structures; *NS* non-significant differences

*P* < 0.05 (*); *P* < 0.01 (**)

Cortical bone thickness of rabbits from the E1 and E2 groups was significantly increased compared to the C group. In addition, statistically significant differences were also identified between the groups E1 and E2, and the groups E1 and E3 (Table 2).

**Table 2:** Cortical bone thickness in adult rabbits from the E1, E2, E3 and C groups

Rabbits group		N	Cortical bone thickness (μm)
E1	(1)	120	1224.78 ± 160.09
E2	(2)	120	1104.79 ± 127.29
E3	(3)	120	1059.01 ± 151.78
C	(4)	120	1055.53 ± 112.34
Games–Howell’s test			1:2^**^; 1:3^**^; 1:4^**^; 2:4^*^

*N* number of measurements

*P* < 0.05 (*); *P* < 0.01 (**)

## Discussion

The results of qualitative histological analysis are in accordance to those of others [24–26]. Primary vascular longitudinal, primary vascular radial, irregular Haversian and/or dense Haversian bone tissues were found in all groups of rabbits. However, exposure to quercetin at levels of 10, 100, and 1000 μg/kg bw three times per week through 90 days leads to changes such as increased bone resorption, lower number of secondary osteons in the compact bone microstructure. These lesions were the most significant in rabbits exposed to the lowest dose of quercetin (10 μg/kg bw; E1 group), although they were also present in the E2 group given a dose of 100 μg/kg bw. The absence of primary vascular longitudinal bone tissue in some areas near the endosteal border and a lower density of secondary osteons in the middle part of the *substantia* *compacta* in these animals influences accelerated bone resorption at the endosteal surface.

Despite an increasing knowledge of quercetin’s beneficial activities as high potential free radical scavenger in vitro, it may also have prooxidant effects under certain conditions [27, 28]. This prooxidant activity can contribute to the generation of ROS [29, 30] which have been shown to stimulate osteoclastic bone resorption [31].

According to Ahlborg et al. [32], excessive bone loss from the endocortical surface induces a mechanical stress on bone tissue, resulting in stimulation of periosteal bone apposition. Deposition of bone tissue onto the periosteal surface is considered to be an adaptive bone response to maintain resistance to bone loss and fractures. In our study, the predominance of periosteal bone apposition over endocortical bone resorption was associated with the thickest layer of primary vascular longitudinal bone tissue (E1 group) and increased thickness of cortical bone in rabbits from the E1 and E2 groups [33, 34]. The lower density of secondary osteons in the middle part of *substantia compacta* in rabbits from the E1, E2 and E3 groups could lead to weakness of biomechanical properties of their bones due to increased formation of microcracks [35].

The results also revealed significantly decreased sizes of the primary osteons’ vascular canals in rabbits from the E2 and E3 groups. Primary osteons’ vascular canals contain blood vessels which provide nutrition for the bone [36]. Pries et al. [37] showed that blood vessels can adapt its structure (vascular remodeling) in response to continuous functional changes. The results of several in vitro studies [38–41] documented the suppressive effect of quercetin on the expression of enzyme nitric oxide synthase (eNOS), which catalyzes a release of nitric oxide (NO), and endothelial cell proliferation. NO acts as a potential vasodilator and decreased production leads to vasoconstriction of blood vessels [42]. Therefore, the reduced size of primary osteons’ vascular canals may be associated with structural changes of blood vessels present in primary osteons due to a negative effect of higher doses of quercetin on the eNOS expression. In addition, vascular endothelial growth factor (VEGF) is considered to play a central role in angiogenesis under pathological conditions [43, 44]. Several studies [45–47] have demonstrated an inhibitory effect of quercetin on the expression of VEGF. Furthermore, the inhibitory effect of quercetin on proliferation, migration and differentiation of endothelial cells during angiogenesis was observed [48, 49]. For this reason, quercetin-induced changes during angiogenesis could contribute to the smaller primary osteons’ vascular canals in rabbits from the E2 and E3 groups. Interestingly, the size of the vascular canals of primary osteons did not change in rabbits from the E1 group. This indicates a dose-dependent effect of quercetin on their size. On the other hand, significantly decreased sizes of the secondary osteons were observed in all groups exposed to quercetin. We assume that evident alterations in the size of secondary osteons in these animals could be related to the destruction of collagen fibers present in the secondary osteons [50]. Kang et al. [51] found that quercetin (6.25–50 µmol/l) inhibited collagen synthesis on keloid-derived fibroblasts in vitro. The negative effect of various concentrations of quercetin (20, 40, and 80 µmol/l) on collagen reduction (more than 50 % in case of the highest dose) in human fibroblasts was also confirmed by Stipcevic et al. [52].

## Conclusions

The study demonstrates that prolonged intramuscular application of quercetin has a significant effect on both qualitative and quantitative histological characteristics of the compact bone in adult female rabbits at doses of 10, 100, and 1000 μg/kg bw. A positive dose–response of quercetin has been identified for the sizes of primary osteons’ vascular canals and secondary osteons. On the contrary, quercetin had a negative dose–response on qualitative histological characteristics of the compact bone and cortical bone thickness. Our study provides initial information related to quercetin’s impact on femoral bone microstructure in rabbits. These findings may be useful for future insights into bone microstructural changes after the application of various nutrients.
